# Результаты клинической апробации системы FreeStyle Libre у детей с сахарным диабетом 1 типа: улучшение гликемического контроля в сочетании со снижением риска тяжелой гипогликемии и диабетического кетоацидоза

**DOI:** 10.14341/probl12877

**Published:** 2022-02-22

**Authors:** Д. Н. Лаптев, О. Б. Безлепкина, Е. С. Демина, О. А. Малиевский, И. Л. Никитина, Ю. Г. Самойлова, В. А. Петеркова

**Affiliations:** Национальный медицинский исследовательский центр эндокринологии; Национальный медицинский исследовательский центр эндокринологии; Российская детская клиническая больница; Башкирский государственный медицинский университет; Национальный медицинский исследовательский центр им. В.А. Алмазова; Сибирский государственный медицинский университет; Национальный медицинский исследовательский центр эндокринологии

**Keywords:** сахарный диабет 1 типа, непрерывный мониторинг глюкозы, гипогликемия, диабетический кетоацидоз

## Abstract

**ОБОСНОВАНИЕ:**

ОБОСНОВАНИЕ. Традиционный самоконтроль глюкозы крови с помощью глюкометров обладает ограниченной информативностью и сопровождается существенным психологическим дискомфортом, особенно у детей. Использование системы флеш-мониторирования глюкозы (ФМГ) — FreeStyle Libre позволяет преодолеть многие барьеры, связанные с измерением глюкозы, и улучшить метаболический контроль.

**ЦЕЛЬ:**

ЦЕЛЬ. Оценить эффективность применения ФМГ у детей с сахарным диабетом 1 типа (СД1) в отношении показателей гликемического контроля, эпизодов тяжелой гипогликемии и диабетического кетоацидоза (ДКА).

**МАТЕРИАЛЫ И МЕТОДЫ:**

МАТЕРИАЛЫ И МЕТОДЫ. Проведено многоцентровое проспективное обсервационное исследование в реальной клинической практике. Всего в исследование были включены 469 пациентов (258 мальчиков и 211 девочек), соответствующих критериям включения. Медиана возраста составила 11,3 (8,4–14,6) года, длительность СД1 — 4,2 (2,1–7,1) года. Длительность наблюдения пациента составляла 6 мес.

**РЕЗУЛЬТАТЫ:**

РЕЗУЛЬТАТЫ. После 3 и 6 мес использования ФМГ показатели HbA1c статистически значимо снизились с 7,4 до 7,1 и 7,2% соответственно (p<0,001). Число детей с уровнем HbA1c ><7,5% увеличилось с 51 до 60 и 58% через 3 и 6 мес соответственно (p><0,001). Частота случаев ДКА и тяжелой гипогликемии, а также доля пациентов с подобными эпизодами были статистически значимо меньше после 6 мес использования ФМГ по сравнению с исходным уровнем (p><0,001). ЗАКЛЮЧЕНИЕ. Клиническая апробация продемонстрировала существенное улучшение метаболического контроля у детей с СД1 после 6 мес использования ФМГ: снижение показателей HbA1c, сопровождающееся увеличением числа детей, достигших целевого показателя, а также значительное уменьшение частоты ДКА и тяжелой гипогликемии.>< 0,001). Число детей с уровнем HbA1c< увеличилось с 51 до 60 и 58% через 3 и 6 мес соответственно (p<0,001). Частота случаев ДКА и тяжелой гипогликемии, а также доля пациентов с подобными эпизодами были статистически значимо меньше после 6 мес использования ФМГ по сравнению с исходным уровнем (p><0,001). ЗАКЛЮЧЕНИЕ. Клиническая апробация продемонстрировала существенное улучшение метаболического контроля у детей с СД1 после 6 мес использования ФМГ: снижение показателей HbA1c, сопровождающееся увеличением числа детей, достигших целевого показателя, а также значительное уменьшение частоты ДКА и тяжелой гипогликемии.>< 0,001). Частота случаев ДКА и тяжелой гипогликемии, а также доля пациентов с подобными эпизодами были статистически значимо меньше после 6 мес использования ФМГ по сравнению с исходным уровнем (p<0,001). ЗАКЛЮЧЕНИЕ. Клиническая апробация продемонстрировала существенное улучшение метаболического контроля у детей с СД1 после 6 мес использования ФМГ: снижение показателей HbA1c, сопровождающееся увеличением числа детей, достигших целевого показателя, а также значительное уменьшение частоты ДКА и тяжелой гипогликемии. >< 0,001).

**ЗАКЛЮЧЕНИЕ:**

ЗАКЛЮЧЕНИЕ. Клиническая апробация продемонстрировала существенное улучшение метаболического контроля у детей с СД1 после 6 мес использования ФМГ: снижение показателей HbA1c, сопровождающееся увеличением числа детей, достигших целевого показателя, а также значительное уменьшение частоты ДКА и тяжелой гипогликемии.

## Abstract (en)

**BACKGROUND:**

BACKGROUND: Self-monitoring of blood glucose (SMBG) with glucometers provides only a snapshot of the glycemic profile and is accompanied by significant psychological discomfort and pain, especially in children. Flash Glucose Monitoring System — FreeStyle Libre (FSL) overcomes many of the barriers associated with glucose measurement and improves metabolic control.

**AIM:**

AIM: To evaluate the efficacy of FSL in children with type 1 diabetes mellitus (T1DM) in terms of glycemic control, episodes of severe hypoglycemia (SH) and diabetic ketoacidosis (DKA).

**MATERIALS AND METHODS:**

MATERIALS AND METHODS. A multicenter, prospective, observational study in real clinical practice was carried out. A total of 469 subjects (258 boys and 211 girls) aged 4–18 were included in the study. The median age was 11.3 (8.4–14.6) years, duration of T1DM — 4.2 (2.1–7.1) years. After FSL start, patient was followed up for 6 months.

**RESULTS:**

RESULTS: After 3 and 6 months of FSL use, HbA1c significantly decreased from 7.4% to 7.1% and 7.2%, respectively (p<0.001). The number of children with HbA1c ><7.5% increased from 51% to 60% and 58% at 3 and 6 months, respectively (p><0.001). The incidence of DKA and SH, as well as the proportion of patients experiencing at least one episode of DKA and SH, were significantly reduced after 6 months of FSL use compared with baseline (p><0.001).

**CONCLUSION:**

CONCLUSION: The Study demonstrated a significant improvement in metabolic control in children with T1DM 6 months after FSL start: decrease in HbA1c, accompanied by reduction in incidence of DKA and SH.

## ОБОСНОВАНИЕ

Система флеш-мониторирования глюкозы (ФМГ) — FreeStyle Libre Flash Glucose Monitoring появилась в Российской Федерации в 2018 г. Основными преимуществами системы являются отсутствие необходимости в калибровке и большая длительность использования датчика. Система ФМГ предоставляет информацию о текущем уровне глюкозы, тенденции (направления и скорости) изменения глюкозы, график глюкозы за последнее и предыдущее время.

Регулярный контроль показателей глюкозы является одним из основных элементов эффективного управления сахарным диабетом 1 типа (СД1) у детей и необходим для достижения и поддержания целевых показателей гликемического контроля для предупреждения формирования осложнений [1–4]. Традиционные методы самоконтроля глюкозы крови (СКГК) с помощью глюкометров обладают ограниченной информативностью и, кроме того, сопряжены с существенным психологическим дискомфортом, особенно у детей. Использование системы ФМГ может позволить преодолеть многие барьеры, связанные с измерением глюкозы и улучшить метаболический контроль у детей с СД1.

В работе представлены результаты клинической апробации системы FreeStyle Libre у детей с СД1.

## ЦЕЛЬ ИССЛЕДОВАНИЯ

Оценить эффективность применения ФМГ у детей с СД1 в отношении показателей гликемического контроля, возникновения тяжелой гипогликемии и диабетического кетоацидоза (ДКА).

## МАТЕРИАЛЫ И МЕТОДЫ

Место и время проведения исследования

Место проведения. Исследование выполнено на базе следующих клинических центров: Национальный медицинский исследовательский центр эндокринологии (Москва), Российская детская клиническая больница (Москва), Башкирский государственный медицинский университет (Уфа), Национальный медицинский исследовательский центр им. В.А. Алмазова (Санкт-Петербург), Сибирский государственный медицинский университет (Томск).

Время исследования. 10.2018–10.2020.

Изучаемые популяции (одна или несколько)

К участию в исследовании были приглашены дети в возрасте >4 и <18 лет с СД1 и уровнем HbA1c менее 10,0% на интенсифицированной инсулинотерапии (путем множественных инъекций — МИИ или непрерывной подкожной инфузии инсулина — НПИИ).

Способ формирования выборки из изучаемой популяции

Отбор пациентов осуществлялся из лиц, обратившихся в соответствующую медицинскую организацию, на основании установленных критериев включения.

Дизайн исследования

Многоцентровое проспективное обсервационное исследование в реальной клинической практике.

Описание медицинского вмешательства (для интервенционных исследований)

Переход на ФМГ осуществлялся во время первой очной консультации и включал обучение правилам установки и использования датчика и сканера, принципам измерения глюкозы и анализа данных. Длительность наблюдения за пациентом составляла 6 мес с момента включения. Исходно при инициации ФМГ и через 3 и 6 мес проводились очные консультации с оценкой общего состояния, исследованием HbA1c, оценкой показателей гликемии, прогресса в отношении целевых показателей гликемического контроля и коррекцией проводимой терапии.

Методы

Исследование уровня HbA1c выполнялось методом жидкостной хроматографии на анализаторе DS5 Glyсomat (DrewSсientific, Нидерланды), методом реакции агглютинации моноклональных антител на анализаторе DCA Vantage Analyzer (Siemens, Германия) или методом высокоэффективной жидкостной хроматографии на анализаторе BioRad D-10 (BioRad Laboratories, США) из образцов сыворотки крови, взятой утром натощак.

Основной исход исследования

Изменение HbA1c и доля пациентов, достигших HbA1c менее 7,5% к 3 и 6-му месяцу исследования по сравнению с исходным уровнем. Изменение частоты случаев ДКА и тяжелой гипогликемии к концу исследования по сравнению с исходным уровнем. Тяжелая гипогликемия определялась как событие с тяжелыми когнитивными нарушениями (включая кому и судороги), требующее помощи другого человека для активного введения углеводов, глюкагона или других корректирующих действий.

Дополнительные исходы исследования

Зависимость уровня HbA1c и показателей времени в диапазонах: ВЦД — время в целевом диапазоне 3,9–10 ммоль/л, ВВД — время выше целевого диапазона >10 ммоль/л, ВНД — время ниже целевого диапазона <3,9 ммоль/л от частоты сканирования.

Статистический анализ

Размер выборки предварительно не рассчитывался. Обработка и анализ статистических данных проводились с использованием TIBCO Software Inc. (2017) Statistica (data analysis software system), version 13 и OpenEpi (Open Source Epidemiologic Statistics for Public Health, Atlanta, GA, USA; http://www.openepi.com). Количественные данные представлены в виде медианы и интерквартильного размаха Me (25–75 перцентиль); качественные данные представлены в виде абсолютных значений (n) и/или частот (%), данные о частоте эпизодов ДКА и тяжелой гипогликемии представлены в виде частоты эпизодов в пересчете на 100 пациентов в год. Различие между количественными непрерывными признаками в зависимых выборках оценивалось с помощью Т-критерия Вилкоксона, в нескольких выборках — с помощью критерия Краскела–Уоллиса. В случае множественных сравнений использовалась поправка Бонферрони. Различие между качественными, номинальными признаками оценивалось с помощью точного критерия Фишера, различие между частотой случаев — с помощью точного критерия Mid-P. Значение р менее 0,05 считалось статистически значимым.

Этическая экспертиза

Протокол исследования одобрен локальным Комитетом по этике ФГБУ «НМИЦ эндокринологии» Минздрава России, протокол №3 от 14.02.2018. До включения в исследования законные представители пациентов подписали информированное согласие на участие в нем.

## РЕЗУЛЬТАТЫ

Всего в исследование были включены 469 пациентов, соответствующих критериям включения. Все пациенты получали инсулинотерапию путем МИИ или НПИИ и осуществляли регулярный СКГК с помощью глюкометров. Клиническая характеристика пациентов и основные результаты исследования обобщены в таблице 1.

**Table:** Таблица 1. Клиническая характеристика пациентов и основные показатели исходно, через 3 и 6 мес после инициации ФМГ. Данные представлены в виде: медиана (интерквартильный диапазон), если не указано иного. Для непрерывных переменных использовался Т-критерий Уилкоксона, для номинальных — двусторонний точный критерий Фишера или Mid-P

Показатель	Исходно	3 мес	P	6 мес	P
Клиническая характеристика (n=469)
Возраст, годы	11,3 (8,4–14,6)	-	-	-	-
Пол, м/ж	224/245	-	-	-	-
Длительность диабета, годы	4,2 (2,1–7,1)	-	-	-	-
НПИИ/МИИ	258/211	-	-	-	-
Длительность НПИИ	1,5 (0,8–3)	-	-	-	-
Частота СКГК (в день)	7 (6–10)	2,5 (2–3,8)	<0,001	2 (1–3)	<0,001
Гликемический контроль
HbA1c, %Пациентов с уровнем <7,5%, n (%)Пациентов с уровнем <7,0%, n (%)	7,4 (6,6–8,4)239 (51)170 (36)	7,1 (6,4–8,0)281 (60)205 (44)	<0,0010,0070,012	7,2 (6,5–8,1)274 (58)188 (40)	<0,0010,0260,127
Острые осложнения
ДКАПациентов с ≥1 эпизодом, n (%)Случаев на 100 пациенто-лет (95% ДИ)	28 (6)1,8 (1,3–2,5)	--	--	1 (0,2)0,4 (0,1–2,4)	<0,0010,047
Тяжелая гипогликемияПациентов с ≥1 эпизодом, n (%)Случаев на 100 пациенто-лет (95% ДИ)	42(9)2,3 (1,7–3,0)	--	--	1(0,2)0,4 (0,1–2,4)	<0,0010,019

Гликемический контроль

Показатели HbA1c статистически значимо снизились после 3 и 6 мес использования пациентами ФМГ, на 0,3 и 0,2% соответственно (p<0,001, табл. 1). Помимо этого, количество детей с уровнем HbA1c<7,5% значимо увеличилось на 18 и 15% через 3 и 6 мес наблюдения соответственно (p<0,001).

Диабетический кетоацидоз и тяжелые гипогликемии

После 6 мес использования ФМГ доля пациентов с зафиксированным по меньшей мере одним эпизодом ДКА была статистически значимо меньше по сравнению с исходным уровнем (p<0,001), то же самое отмечалось в отношении частоты случаев ДКА, которая уменьшилась на 77%, в 4,3 раза по сравнению с СКГК (p=0,007).

Доля пациентов с зарегистрированным эпизодом тяжелой гипогликемии также была значимо меньше после 6 мес использования ФМГ (p<0,001), а частота случаев тяжелой гипогликемии снизилась на 82%, в 5,4 раза по сравнению с СКГК (p<0,001).

Частота сканирования и СКГК

Частота СКГК при использовании ФМГ значимо сократилась и составила 2,5 и 2 раза в сутки на 3 и 6-м месяце наблюдения соответственно по сравнению с 7 измерениями до использования ФМГ (p<0,001). Во время исследования пациенты сканировали датчик от 5 до 30 раз в течение дня, что в среднем составило 16,3 раза в сутки.

Большая частота сканирований пациентами сопровождалась более низкими показателями HbA1c, а также большим ВЦД 3,9–10,0 ммоль/л (p<0,001), меньшим временем ВВД >10 ммоль/л (p<0,001), при этом частота сканирований значимо не влияла на ВНД <3,9 ммоль/л. (рис. 1, 2).

**Figure:**
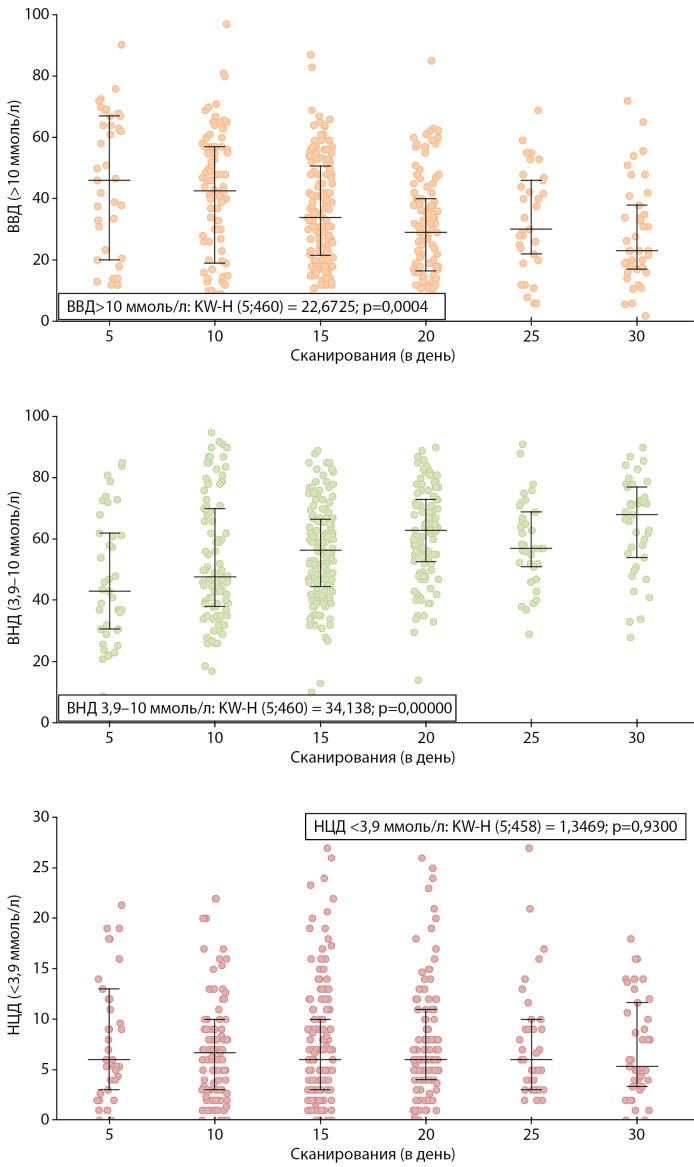
Рисунок 1. Показатели времени в диапазонах в зависимости от частоты сканирования датчика за сутки. ВЦД — время в целевом диапазоне 3,9–10 ммоль/л, ВВД — время выше целевого диапазона >10 ммоль/л, ВНД — время ниже целевого диапазона <3,9 ммоль/л. Данные представлены в виде: медиана (интерквартильный диапазон) и отдельные значения. Использован критерий Краскела–Уоллиса.

**Figure:**
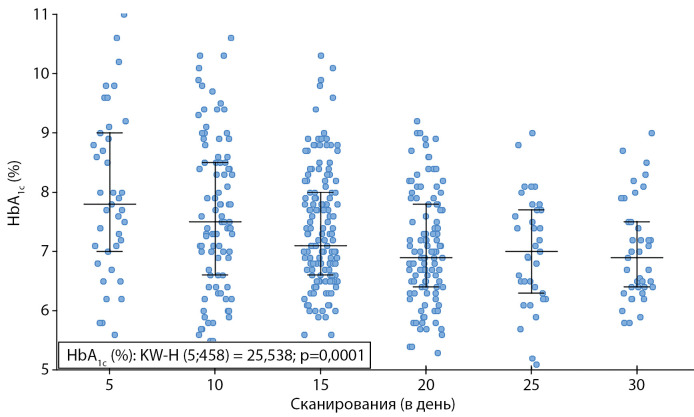
Рисунок 2. Уровень HbA1c в зависимости от частоты сканирования датчика за сутки. Данные представлены в виде: медиана (интерквартильный диапазон) и отдельные значения. Использован критерий Краскела–Уоллиса.

После начала использования ФМГ потребность в использовании глюкометра существенно уменьшилась — частота СКГК статистически значимо сократилась в 3,5 раза (p<0,001).

## ОБСУЖДЕНИЕ

Результаты нашего длительного наблюдения за детьми с СД1 в условиях реальной клинической практики указывают на то, что регулярное использование ФМГ сопровождается значительным улучшением метаболического контроля. Нами продемонстрировано снижение показателей HbA1c, сопровождающееся увеличением числа детей, достигших целевого показателя, а также существенное снижение частоты ДКА и тяжелой гипогликемии после 6 мес использования ФМГ. Как показано другими проведенными к настоящему времени исследованиями, преимуществами технологии непрерывного мониторирования глюкозы являются улучшение НbА1с, снижение частоты и времени гипогликемии и увеличение ВЦД.

Эффективность системы ФМГ в отношении гликемического контроля у детей с СД1 продемонстрирована рядом исследований. В проспективном исследовании SELFY [5] у детей с СД1 в возрасте 4–17 лет (n=76) показано, что после 8 нед использования ФМГ отмечалось снижение HbA1c на 0,4% от исходного уровня (p<0,0001), а ВЦД возросло на 0,9 ч в сутки (р=0,005). У детей с СД1 из регистра Diabetes Prospective Follow-up (DVP) после 12 мес использования ФМГ зарегистрировано снижение среднего уровня HbА1c на 0,1% (р<0,0001) от исходных значений [6]. В наблюдательном исследовании Isabel Leiva-Gea и соавт. [7], проведенном у детей 4–18 лет (n=145) в Испании, было показано, что в группе пациентов с недостаточным уровнем гликемического контроля (средний исходный HbA1c 9,7%) через 3 мес от начала применения ФМГ отмечалось снижение HbA1c на 1,96% (р=0,04).

Влияние применения ФМГ на гликемический контроль во много связано с частотой использования системы. В исследовании Suzuki J. и соавт. [8] показаны положительная корреляция частоты сканирований с продолжительностью ВЦД (r=0,719; P<0,0001) и обратная корреляция с временем ВВД (r=-0,743; P<0,0001), средним уровнем глюкозы и уровнем HbA1c и расчетным значением HbA1c (r=-0,765; -0,815–-0,793 соответственно; P<0,0001) [8].

В отношении частоты острых осложнений СД1 также продемонстрировано преимущество ФМГ по сравнении с традиционным СКГК. В результате проспективного исследования применения ФМГ у детей и молодых людей в возрасте 4–20 лет (n=334) c СД1 в условиях реальной практики было показано, что использование ФМГ в течение 12 мес позволяет уменьшить число эпизодов тяжелой гипогликемии на 53% в сравнении с СКГК (р=0,012) [9]. У детей с СД1 из регистра DVP на фоне применения ФМГ было отмечено сокращение частоты ДКА на 50% (р=0,0254), частоты тяжелых гипогликемий на 23,5% (р=0,0366) [6]. В исследовании Suzuki J. и соавт. [8] общая частота эпизодов гипогликемии 3-й степени при расчете на 100 пациенто-лет сократилась с 4,2 до 0,2 эпизода через 12 мес использования ФМГ. При оценке зависимости частоты эпизодов гипогликемии от средней частоты сканирований в сутки было показано, что наименьшая частота гипогликемий 1 и 2-й степени отмечалась у пациентов с частотой ежедневных сканирований >10 раз (p=0,05) [7].

Применение ФМГ способствует повышению приверженности к контролю уровня глюкозы и качества жизни. По данным Deeb A. и соавт. [10], во время применения ФМГ отмечено статистически значимое увеличение частоты измерения глюкозы: средняя частота сканирований составила 11,6 раза в сутки, в то время как в тот период времени, когда СКГК осуществлялся при помощи глюкометров, его средняя частота составляла 2,87 раза в сутки (p<0,001) [10]. В исследовании SELFY [5] СКГК при помощи глюкометра сократился с 7,7±2,5 до 1,6±1,9 раза в сутки, а частота измерения глюкозы с использованием ФМГ составила 12,9 раз в сутки, при этом применение ФМГ ассоциировалось с улучшением показателей общей удовлетворенности терапией у подростков с СД1 и их родителей при оценке по опроснику The Diabetes Treatment Satisfaction Questionnaire (DTSQ) (р<0,0001). Также на фоне применения ФМГ в течение 3 мес отмечаются уменьшение тревожности, связанной со страхом развития гипогликемии (р=0,0001), повышение качества жизни (р=0,002), снижение уровня HbA1с в среднем на 0,66% (р=0,008), а также уменьшение частоты возникновения эпизодов гипогликемии (р=0,023) от исходных значений [11]. По данным Vergier J. [12], дети с СД1, применявшие ФМГ (n=347), отмечали, что использование данной технологии для осуществления контроля уровня глюкозы позволяет не только избегать проколов пальцев (85,9% пациентов), но и получать больше информации об уровне глюкозы, в частности, в ночной период (60,4% пациентов), легче изменять свои привычки и корректировать образ жизни (89,5% пациентов), чаще определять свой уровень глюкозы (70,6% пациентов).

## ЗАКЛЮЧЕНИЕ

Применение ФМГ у детей в возрасте от 4 до 18 лет с СД1 имеет следующие клинические преимущества по сравнению с СКГК:

Эффективность использования ФМГ во многом зависит от интенсивности и времени использования системы: большая частота сканирований способствует увеличению времени в целевом диапазоне и снижению уровня HbA1c.

## ДОПОЛНИТЕЛЬНАЯ ИНФОРМАЦИЯ

Источники финансирования. Работа выполнена в рамках клинической апробации «Оказание специализированной медицинской помощи детям и подросткам с сахарным диабетом 1 типа с использованием системы Flash-мониторинга глюкозы».

Конфликт интересов. Авторы декларируют отсутствие явных и потенциальных конфликтов интересов, связанных с публикацией настоящей статьи.

Участие авторов. Петеркова В.А. — научное руководство, дизайн и планирование исследования; Лаптев Д.Н. — сбор, анализ и статистическая обработка полученных данных, формирование регистра пациентов, написание и редактирование текста; Демина Е.С. — сбор и обработка полученных данных, формирование и ведение регистра пациентов, редактирование текста; Малиевский О.А. — сбор и обработка полученных данных, формирование и ведение регистра пациентов, редактирование текста; Никитина И.Л. — сбор и обработка полученных данных, формирование и ведение регистра пациентов, редактирование текста; Самойлова Ю.Г. — сбор и обработка полученных данных, формирование и  ведение регистра пациентов, редактирование текста; Безлепкина О.Б. — научное руководство, дизайн и планирование исследования. Все авторы одобрили финальную версию статьи перед публикацией, выразили согласие нести ответственность за все аспекты работы, подразумевающую надлежащее изучение и решение вопросов, связанных с точностью или добросовестностью любой части работы.
